# Searching for novel multimodal treatments in oligometastatic pancreatic cancer

**DOI:** 10.1186/s12885-020-06718-3

**Published:** 2020-03-30

**Authors:** D. M. Filippini, E. Grassi, A. Palloni, R. Carloni, R. Casadei, C. Ricci, C. Serra, G. Ercolani, G. Brandi, M. Di Marco

**Affiliations:** 1 Department of Experimental, Diagnostic and Specialty Medicine University of Bologna, Sant’Orsola-Malpighi Hospital, Massarenti Street 11, 40100 Bologna, Italy; 2 Department of Medical and Surgical Sciences, University of Bologna, Sant’Orsola-Malpighi Hospital, Bologna, Italy; 3 Department of Organ Failure and Transplantation, Ultrasound Unit, Sant’Orsola-Malpighi Hospital, Bologna, Italy; 4 General and Oncologic Surgery, Morgagni-Pierantoni Hospital, AUSL Romagna, Forlì, Italy

**Keywords:** Pancreatic cancer, Oligometastatic cancer, Metastasectomy, Multimodal treatment

## Abstract

**Background:**

Metastatic pancreatic cancer has a median overall survival of less than 12 months, even if treated with chemotherapy. Selected patients with oligometastatic disease could benefit from multimodal treatments connecting chemotherapy and surgical treatment or radiofrequency ablation (RFA) of metastases.

**Case presentation:**

We present a patient with oligometastatic pancreatic cancer recurrence who was successfully treated with a multimodal therapeutic approach.

A 57-year-old male initially presenting with resectable pancreatic cancer underwent pancreatoduodenectomy. The histopathological diagnosis revealed ductal pancreatic adenocarcinoma with positive surgical resection margins and negative lymph nodes. He completed six cycles of adjuvant therapy with gemcitabine (1000 mg/mq 1,8,15q 28), followed by external radiotherapy (54 Gy in 25 fractions) associated with gemcitabine 50 mg/mq twice weekly. Three years later, the patient developed multiple liver metastases, and he started FOLFIRINOX (oxaliplatin 85 mg/mq, irinotecan 180 mg/mq, leucovorin 400 mg/mq and fluorouracil 400 mg/mq given as a bolus followed by 2400 mg/mq as a 46 h continuous infusion,1q 14) as a first-line treatment.

The CT scan showed a partial response after 6 cycles. After multidisciplinary discussion, the patient underwent a laparotomic metastasectomy of the three hepatic lesions. After additional postsurgical chemotherapy with 4 cycles of the FOLFIRINOX schedule, the patient remained free of recurrence for 12 months. A CT scan showed a new single liver metastasis, which was treated with radiofrequency ablation (RFA). A second radiofrequency ablation was performed when the patient developed another single liver lesion 12 months after the first RFA; currently, the patient is free from recurrence with an overall survival of 6 years from the diagnosis.

**Conclusions:**

Our case has benefited from successful multimodal treatment, including surgical and local ablative techniques and systemic chemotherapy. A multimodal approach may be warranted in selected patients with oligometastatic pancreatic cancer and could improve overall survival. Further research is needed to investigate this approach.

## Background

Approximately 49.5% of patients present distant metastases at the time of a pancreatic cancer diagnosis. The median OS of metastatic pancreatic ductal adenocarcinoma (PDAC) is less than 12 months if treated with chemotherapy [1].

Patients with metastatic disease have traditionally been considered as having unresectable disease according to the National Comprehensive Cancer Network treatment guidelines [2].

The liver is a frequent disseminate site for metastatic pancreatic adenocarcinoma followed by the lungs, abdominal lymph nodes, peritoneum/omentum, and adrenal glands [3, 4].

The treatment of choice in metastatic settings is systemic chemotherapy, and the limited data on the possible role of liver surgery in the management of metastatic disease has discouraged resection. Although hepatic resection has been well established in other gastrointestinal malignancies, such as colorectal cancer and neuroendocrine tumours with liver metastases, metastasectomies for PDAC remain highly controversial and seem to offer no benefit for the majority of cases. Nevertheless, metastasectomy could be the new treatment option to improve survival in a highly selected group of patients, particularly those with oligometastatic pancreatic cancer [5].

T. Hackert et al. published the largest series of resected metastatic PDACs, which showed a long-term survival benefit of 10% in selected patients who received liver or interaortocaval lymph node metastases resection compared to the reported survival time after palliative treatment alone [6].

Therefore, we report the case of one selected patient affected by PDAC in stage IV due exclusively to hepatic metastases who benefited from surgical treatment of the liver metastases in a multimodal context, including local ablative techniques and systemic chemotherapy.

## Case presentation

A 57-year-old Caucasian, ECOG PS 0 male patient, was referred to our hospital to be examined and treated for a resectable cancer of the hooked process of the pancreas. The levels of carbohydrate antigen 19–9 (CA19–9) and carcinoembryonic antigen (CEA) were 50 U/ml (normal value < 37.0) and 1.2 ng/ml (normal value < 5 ng/ml), respectively.

Therefore, in July 2013, pancreatoduodenectomy and regional lymphadenectomy were performed. The histopathological diagnosis revealed pancreatic adenocarcinoma of ductal origin with moderately differentiated mucinous aspects, G2, pT3 pN0 (0/30) Mx (AJCC 8th revised edition). The surgical resection margins were positive. The margin involved was the superior mesenteric artery (SMA). There were no post-operative complications. The CA19–9 value (8 U/ml) was negative. The patient underwent six cycles of adjuvant chemotherapy with gemcitabine 1000 mg/mq 1,8,15 q28, followed by chemoradiotherapy. Radiotherapy consisted of an external beam treatment administered to the pancreatic bed with a total dose of54 Gy in 25 fractions associated with concurrent radiosensitizer bi-weekly gemcitabine 50 mg/mq.

**Fig. 1:**
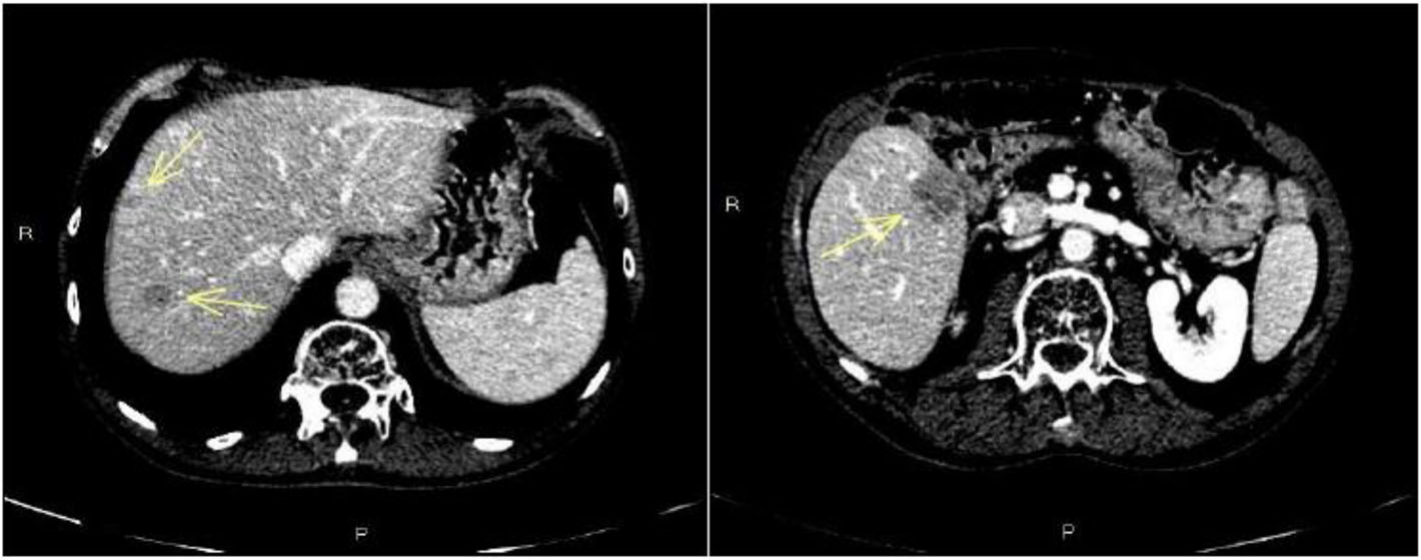
The abdomen CT scan highlighted the appearance of some secondary hepatic lesions (yellow arrows)

Subsequently, the patient underwent follow-up, which was negative for 3 years until a computerized tomography (CT) scan showed at least six secondary hepatic lesions, as shown in Fig. 1. Liver metastases were located exactly at hepatic segments VIII, VII, V-VI, V, IV and II.

The CA19–9 value was > 900 U/ml, and the CEA was negative.

The patient started the first-line treatment with six courses of FOLFIRINOX: oxaliplatin 85 mg/mq, irinotecan 180 mg/mq, leucovorin 400 mg/mq and fluorouracil 400 mg/mq given as a bolus followed by 2400 mg/mq as a 46 h continuous infusion,1q 14; the patient required a 20% dose reduction and prophylactic granulocyte colony stimulating factor (G-CSF) for grade 4 neutropenia.

After six cycles of chemotherapy, the re-evaluation CT scan showed only three liver lesions with increased (18)F-FDG PET uptake. Multiple lymphadenopathy masses measuring 1.5 cm, without focal uptake on the (18)F-FDG PET, were observed; these lesions also had disappeared at the subsequent re-evaluations, hence they were not considered pathological lymphadenopathies.

The case was discussed in a multidisciplinary context; in consideration of the haematological toxicity (persistent grade 4 neutropenia), despite good gastroenterologic tolerability, optimal performance status, the partial response of the disease and its exclusive hepatic localization, the patient was judged suitable for surgery. The patient underwent a laparotomic wedge resection (without intraoperative ultrasound) of the three remaining hepatic lesions localized at hepatic segment VIII, VII and V.

The post-operative course was characterized by intermittent fever associated with positive blood cultures and the appearance of fluid collection, which resolved with antibiotic therapy and drainage of the post-operative collection.

The histopathological examination confirmed hepatic metastases from adenocarcinoma with aspects of poor differentiation compatible with pancreatic origin; the surgical resection margins were negative. In consideration of the patients’ good general condition, and even though there was no clear evidence of a post-metastasectomy chemotherapy role, after collegial discussion, we decided to resume chemotherapy at further reduced doses, given the previously observed haematological toxicity.

Afterwards, the patient was treated for two more months with FOLFIRINOX (oxaliplatin 85 mg/mq, irinotecan 180 mg/mq, leucovorin 400 mg/mq and fluorouracil 400 mg/mq given as a bolus followed by 2400 mg/mq as a 46-h continuous infusion, 1q 14)*.*

At the end of chemotherapy, CEA and CA19–9 were at normal levels.

**Fig. 2:**
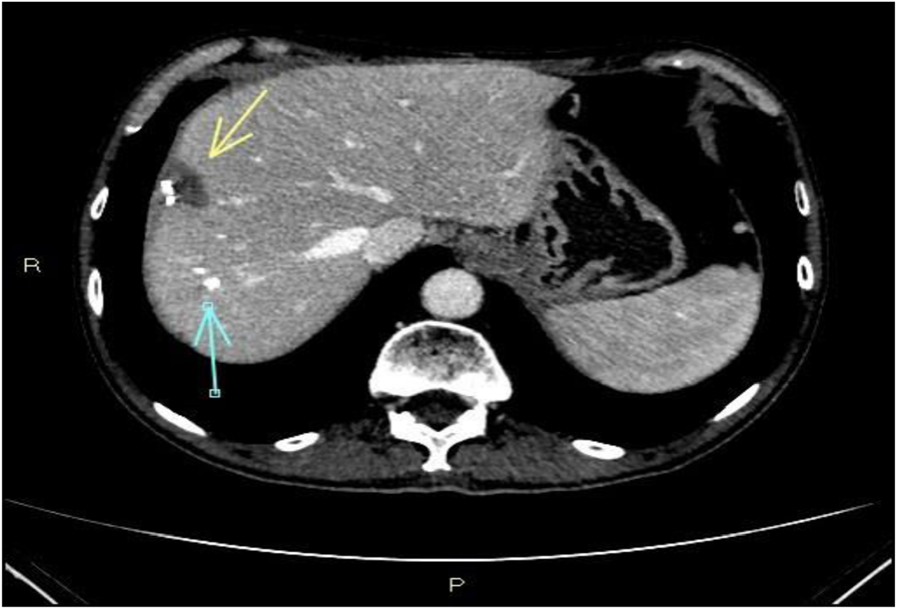
This CT scan image represents the results of the wedge resection and radiofrequency ablation (RFA) of various hepatic lesions. The yellow arrow represents a fluid hepatic collection as a result of radiofrequency. The blue arrow indicates a clip as a result of wedge resection

Seven months after the end of chemotherapy, there was evidence of liver recurrence at the S8 level, which was confirmed by contrast-enhanced ultrasound (CEUS) and magnetic resonance imaging (MRI); a lesion of 1,4 × 1,2 cm was identified (Fig. 2). A biopsy of this hepatic lesion was performed, and the histological test showed metastases from pancreatic adenocarcinoma.

There were no complications after CEUS and subsequent biopsy. CEA and CA19–9 were negative.

After multidisciplinary discussion, in consideration of the previous post-surgical septic complication and according to the patient’s will (he refused both systemic chemotherapy and surgical approach), radiofrequency ablation of the lesion was performed. This was performed via the ultrasound-guided route through two needle insertions (maximum power 140 W, 12 and 6 min, respectively); on the subsequent CEUS, there was complete necrosis of the lesion. Afterwards, the patient underwent follow-up. After 12 months from the first RFA, CT scan showed secondary abnormalities at the V-segment with a maximum size of 1.8 × 1.1 cm; the lesion was heterogeneously vascularized in the arterial phase. There was a slight rise in CA19–9 (32.5 U/ml) but the value was still within the normal range; CEA was also negative.

Biopsy with a Biomol 18-G needle was performed, and the histological examination was indicative of a metastatic lesion of pancreatic origin. Therefore, the patient underwent radiofrequency thermoablation of the lesion at the V hepatic segment measuring 1.9 cm.

Radiofrequency thermotherapy treatment was carried out with an exposed tip of 13 cm and length of 20 cm, with 1 needle insertion on the nodule of the V segment (maximum power 147 W for 11 min).

The post-procedure course was regular, and the ultrasound check after 1 day showed necrotic outcomes without complications.

**Fig. 3:**
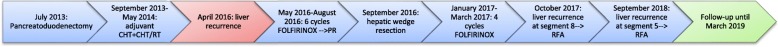
Clinical time course of the treated patient

At the date of the last follow-up (March 2019), the patient was in excellent general conditions and free from recurrence with an overall survival of 6 years from the diagnosis. CEA and CA19–9 were negative. In the Fig. 3 we resumed the clinical time course of the patient.

## Discussion and conclusions

Despite the poor prognosis of pancreatic cancer, we report the case of a pancreatic cancer patient with liver recurrence who was successfully treated with a multimodal approach, including chemotherapy, metastasectomy and radiofrequency ablation of the liver lesions.

The efficacy of a multimodal approach in the management of liver recurrence from pancreatic cancer lacks the support of conclusive data from prospective trials, but for selected patients with only liver metastases and indolent disease course, this approach might represent a reasonable option to improve their overall survival.

PDAC metastases are most commonly observed in the liver, followed by the lungs, peritoneum/omentum, and adrenal glands [3, 7].

The presence of hepatic metastases is indicative of a more aggressive prognosis than lung metastases.

In fact, as reported in a retrospective analysis conducted by Ibrahim H. Sahin et al. [8], patients with oligometastatic disease in the lung could receive a less intensified treatment, (e.g., single agent or doublet therapy as opposed to triplet therapy), whereas patients with liver lesions may benefit from a more aggressive treatment approach. These observations could be partly explained by the heterogeneity of PDAC and of the homing microenvironment.

The treatment options for PDAC liver recurrence might be different and include systemic chemotherapy, surgery (hepatic resection) and local ablative techniques (embolization, transcatheter arterial chemoembolization and radiofrequency ablation) [1].

The evidence available thus far is derived from retrospective analyses with small sample sizes, which have mostly focused on synchronous liver resections during pancreatectomy rather than on the treatment of cancer recurrence.

The increasing safety of surgery in recent decades, with mortality rates of < 5%, has led to an extension of localized approaches in pancreatic cancer. Surgical techniques and outcomes following surgical resection have evolved over the years, but in the management of metastatic pancreas cancer, there is still controversy about metastasectomy and local ablative techniques [9].

Recently, Kandel et al. performed a case-control study to demonstrate better survival and outcomes of an extremely selected group of patients with PDAC with oligometastases (lung or liver) treated with neoadjuvant chemotherapy, metastasectomy and/or RFA, and primary tumour resection compared with non-metastases resected patients [5].

Results from the pooled analysis of 11 cohort studies with 1147 patients conducted by Yu and colleagues showed that surgical resection of hepatic metastases can be performed safely for all pancreatic cancer patients with liver metastases. For the surgical group, the median 1-year, 3-year and 5-year survival rates was 40.9, 13.3, and 2.9%, respectively, with a median survival of 9.9 months. Surgical resection of hepatic metastases was associated with significantly improved overall 1-year and 3-year survival (*p* < 0.001) [10].

Another important issue concerns the role of induction chemotherapy regimens, such as FOLFIRINOX and nab-paclitaxel, used before a possible surgery of metastases; this approach is reserved for only a minority of subjects experiencing significant downstaging, influencing OS. Nevertheless, the role of preoperative chemotherapy in this setting has not been investigated, and evidence-based strategies are lacking.

Data from other studies exploring this approach have indicated that metastasectomy can be performed safely and with a survival benefit in some patients, but the selection of patient is critical to any pursuit of surgical management [6, 11–14].

To date, the identification of the best selection criteria to identify patients who could benefit from metastasectomy in the setting of oligometastatic disease needs to be validated. Nevertheless, in our opinion, various clinical factors deserve consideration in the selection of patients for metachronous metastases surgical resection, including a good performance status, a limited number of liver metastases (≤ 3) (in compliance with the criteria used for performing liver metastasectomy in colon cancer), an indolent disease course, chemosensitivity of the metastases to “neoadjuvant” chemotherapy, a high possibility to gain a resection R0 after metastasectomy and a high-volume cancer centre in which to perform the surgery.

Prospective studies are required to clarify the potential therapeutic utility and operative indications of liver metastasectomy in the setting of modern interdisciplinary management of PDAC.

Notably, the median overall survival reported after surgical resection of liver metastases is not very different from that in recent trials of modern chemotherapy regimens in metastatic settings [15]. Therefore, the winning strategy could be to combine systemic and loco-regional treatments, including metastasectomy, in a subset of highly selected patients affected by metachronous metastases from PDAC.

In our case, the “selected” patient received survival benefits from multimodal treatments (chemotherapy, metastasectomy, radiofrequency ablation) of hepatic metachronous metastasis from pancreatic ductal adenocarcinoma. In conclusion, careful clinical judgement and prudent selection of patients are mandatory as part of a multidisciplinary approach. Further studies in selected patients are needed to investigate this approach.

## Data Availability

Data used in the current manuscript are available from the corresponding author on reasonable request.

## Abbreviations

RFA: Radiofrequency ablation
FOLFIRINOX: Oxaliplatin, irinotecan, leucovorin and fluorouracil
CT: Computerized tomography
PDAC: Pancreatic ductal adenocarcinoma
ECOG PS: Eastern Cooperative Oncology Group performance status
CEA: Carcinoembryonic antigen
CA19–9: Carbohydrate antigen 19–9
G-CSF: Granulocyte colony stimulating factor
CEUS: Contrast-enhanced ultrasound
MRI: Magnetic resonance imaging
OS: Overall survival
CHT: Chemotherapy
RT: Radiotherapy
SMA: Superior mesenteric artery
